# eHealth-Based Behavioral Intervention for Increasing Physical Activity in Persons With Multiple Sclerosis: Fidelity Protocol for a Randomized Controlled Trial

**DOI:** 10.2196/12319

**Published:** 2019-03-01

**Authors:** Stephanie L Silveira, Justin McCroskey, Brooks C Wingo, Robert W Motl

**Affiliations:** 1 Exercise Neuroscience Research Lab Department of Physical Therapy University of Alabama at Birmingham Birmingham, AL United States; 2 Department of Occupational Therapy University of Alabama at Birmingham Birmingham, AL United States

**Keywords:** fidelity, multiple sclerosis, behavior change, exercise

## Abstract

**Background:**

The rate of physical activity is substantially lower in persons with multiple sclerosis (MS) than in the general population. This problem can be reversed through rigorous and reproducible delivery of behavioral interventions that target lifestyle physical activity in MS. These interventions are, in part, based on a series of phase II randomized controlled trials (RCTs) supporting the efficacy of an internet-delivered behavioral intervention, which is based on social cognitive theory (SCT) for increasing physical activity in MS.

**Objective:**

This paper outlines the strategies and monitoring plan developed based on the National Institutes of Health Behavior Change Consortium (NIH BCC) treatment fidelity workgroup that will be implemented in a phase III RCT.

**Methods:**

The Behavioral Intervention for Physical Activity in Multiple Sclerosis (BIPAMS) study is a phase III RCT that examines the effectiveness of an internet-delivered behavioral intervention based on SCT and is supported by video calls with a behavioral coach for increasing physical activity in MS. BIPAMS includes a 6-month treatment condition and 6-month follow-up. The BIPAMS fidelity protocol includes the five areas outlined by the NIH BCC. The study design draws on the SCT behavior-change strategy, ensures a consistent dose within groups, and plans for implementation setbacks. Provider training in theory and content will be consistent between groups with monitoring plans in place such as expert auditing of calls to ensure potential drift is addressed. Delivery of treatment will be monitored through the study website and training will focus on avoiding cross-contamination between conditions. Receipt of treatment will be monitored via coaching call notes and website monitoring. Lastly, enactment of treatment for behavioral and cognitive skills will be monitored through coaching call notes among other strategies. The specific strategies and monitoring plans will be consistent between conditions within the constraints of utilizing existing evidence-based interventions.

**Results:**

Enrollment began in February 2018 and will end in September 2019. The study results will be reported in late 2020.

**Conclusions:**

Fidelity-reporting guidelines provided by the NIH BCC were published in 2004, but protocols are scarce. This is the first fidelity-monitoring plan involving an electronic health behavioral intervention for increasing physical activity in MS. This paper provides a model for other researchers utilizing the NIH BCC recommendations to optimize the rigor and reproducibility of behavioral interventions in MS.

**Trial Registration:**

ClinicalTrials.gov NCT03490240; https://www.clinicaltrials.gov/ct2/show/NCT03490240.

**International Registered Report Identifier (IRRID):**

DERR1-10.2196/12319

## Introduction

Physical activity is low among adults in the United States and even lower among persons with multiple sclerosis (MS) [1,2]. This lower rate of physical activity is important because persons with MS might experience greater benefits from physical activity than the general population [3] if we can change this behavior. The standard approach for promoting physical activity in MS involves structured, supervised exercise training [4] and has resulted in considerable benefits [3], but the low rate of physical activity in people with MS has not changed over the past 25 years [1]. Researchers have recently advocated moving away from structured exercise training and focusing on behavioral interventions for changing the physical activity lifestyle in MS [5,6]. Such behavioral interventions teach people skills, techniques, and strategies for changing physical activity (ie, behavior-change techniques), which are typically based on a health behavior theory [5,6], and can be delivered through electronic health (eHealth; ie, provision of health-related services through the internet or related technologies). EHealth addresses major barriers to physical activity in persons with MS, namely, transportation and cost [7].

We recently completed a 6-month, phase II, randomized controlled trial (RCT) that examined the efficacy of a newly developed internet website that delivered information on behavior-change techniques aligned with social cognitive theory (SCT) [8] by using electronic learning (e-learning) for increasing physical activity and improving symptoms, walking impairment, and neurological disability [9]. Bandura’s SCT is an evidence-based learning theory that highlights the unique role of observing behavior and increasing knowledge for effectively changing behavior through interactions between the person and the environment [8]. E-Learning is the process of extending learning and delivering instructional materials using digital media (eg, interactive videos through the internet) [10]. Participants with MS (N=47) were randomly assigned to the e-learning, behavioral intervention (n=23) or control (n=24) condition. Outcome assessments were administered before and after the 6-month study period. There were positive intervention effects on self-reported and objectively measured moderate-to-vigorous physical activity (MVPA) as well as fatigue, depression, and anxiety symptoms; walking mobility; and disability status. We included a small battery of fidelity metrics in that study. Compliance with weekly step-count entry was 99% and that with weekly coaching video chat sessions was 96%. Such evidence served as proof of principle for the design of a planned phase III RCT testing the effectiveness of this approach for improving physical activity and secondary outcomes as well as examining mediators based on SCT (eg, self-efficacy or goal setting) [11]. This phase III intervention fits within the evaluation phase of the Medical Research Council Framework for assessing the effectiveness of the intervention and understanding change processes [12], which is further described in the recently published protocol focusing on primary, secondary, and tertiary outcomes [11].

The undertaking of a phase III RCT requires an additional protocol and method for monitoring fidelity; yet, fidelity methods are infrequently and inconsistently reported for nonpharmacological intervention trials, in general [13]. We are not aware of any reported fidelity protocols for eHealth-based behavioral interventions aimed to increase physical activity in MS. Fidelity refers to the degree to which an intervention is delivered as planned or intended [13] and addresses both the internal and external validity of a study and its outcomes. This is pertinent in all phases of research, particularly phase III RCTs, wherein factors such as varying treatment dose and provider training can influence intervention delivery and study outcomes.

The National Institutes of Health Behavior Change Consortium (NIH BCC) treatment fidelity workgroup published recommendations for incorporating treatment fidelity practices into health behavior research [13]. These recommendations focus on five areas: study design, provider training, delivery of treatment, receipt of treatment, and enactment of treatment [13]. These five areas include goals, descriptions, and sample strategies that help guide researchers toward conducting rigorous research that is reproducible. The NIH BCC recommendations were published in 2004, and some studies published since have measured theoretical fidelity (ie, SCT), which is a primary concern in fidelity monitoring because the efficacy of programs depends on effects from the specific behavior-change foundations grounded in theory rather than extraneous variables [14,15]. Additionally, facilitator-specific adherence is emphasized in another physical activity intervention [16]. However, one recent literature review indicated that there was still little uniformity in the definition and implementation of fidelity protocols [17]. This is disappointing and supports the need for greater attention toward fidelity in the development and execution of interventions, but few researchers provide this information in study protocols and publications, particularly for MS.

This paper describes the fidelity protocol for the Behavioral Intervention for Physical Activity in Multiple Sclerosis (BIPAMS) study based on the five areas identified by the NIH BCC. Such a protocol paper is essential to clearly document our fidelity metrics and approaches (ie, rigor and reproducibility) and offer a guide for other researchers conducting eHealth behavioral interventions to change physical activity in persons with MS.

## Methods

### Overview and Participants

BIPAMS is a phase III RCT that will test the effectiveness of a behavioral intervention [11] for increasing physical activity and improving secondary outcomes in a large sample of people with MS residing in the United States. The primary outcome is accelerometry as an objective measure of minutes/day of MVPA over a 7-day period. The secondary outcomes are self-reported measures of physical activity, walking mobility, cognition, fatigue, depression, anxiety, pain, sleep quality, and quality of life. The tertiary outcomes are mediator variables (eg, self-efficacy) based on SCT. We will recruit a sample of 280 persons with MS from across the United States through postal and electronic advertisements delivered using the National MS Society, North American Research Center on Multiple Sclerosis, and iConquerMS. We will further distribute advertisements in the MS Centers identified through the National Multiple Sclerosis Society website and request that the materials be distributed among persons living with MS who visit the centers for services. The advertisements will describe the study as one comparing two different approaches delivered through the internet for managing the consequences of MS and improving health indicators. Those interested in participation will contact the study project coordinator either by email or telephone; we will establish a toll-free telephone number owing to the nationwide recruitment effort. This initial email or telephone call will be followed up by a phone call from the project coordinator who will describe the study and its procedures, answer all questions, and conduct a screening for inclusion criteria. The inclusion criteria involve diagnosis of MS; free of relapse in the past 30 days; internet and email access; willingness to complete the questionnaires, wear the accelerometer, and undergo randomization; inactive status defined as not engaging in regular physical activity (30 minutes accumulated per day) on more than 2 days of the week during the previous 6 months; ability to ambulate with or without assistance (ie, walk with or without a cane or walker, but not a wheelchair); and age between 18 and 64 years. We will exclude all individuals with moderate or high risk for undertaking strenuous or maximal exercise using the Physical Activity Readiness Questionnaire (PAR-Q) [15]. During the initial phone contact with the project coordinator, participants will verbally respond to the PAR-Q, and those individuals who report no more than one Yes or affirmative response on the seven items on the PAR-Q will be considered at low risk and included for participation. All other individuals will be considered at moderate or high risk and excluded from participation and further advised to seek medical guidance before becoming more physically active. Participants (N=280) will be randomized into the behavioral intervention condition (BIPAMS; n=140) or a social contact, attention control condition focused on general wellness (WellMS; n=140).

### Behavioral Intervention for Physical Activity in Multiple Sclerosis Intervention Protocol

As described previously [9], the BIPAMS behavioral intervention consists of two primary components, namely, a dedicated internet website and one-on-one video calls with a behavioral coach for increasing physical activity. The WellMS control condition provides an internet website and one-on-one video calls with a behavioral coach for discussion about self-managing MS symptoms through health behaviors other than physical activity (eg, diet and nutrition). A comparison of conditions is presented in Table 1. The conditions will be administered over 6 months and supported by trained behavioral coaches who will be uninvolved in screening, recruitment, random assignment, and outcome assessment. The coaches meet with participants on “content weeks” via one-on-one video calls using Skype (Microsoft Corp, Luxembourg); this starts with weekly calls and modules that taper off in frequency over time for both conditions. There is a 6-month follow-up period wherein participants will not access the study website or engage in video calls with behavior coaches. We will collect primary, secondary, and tertiary outcome data every 6 months over the 12-month period (ie, baseline, immediate follow-up, and 6-month follow-up). This study has been approved by an Institutional Review Board, and the trial is registered at ClinicalTrials.gov (NCT03490240).

### Study Fidelity Protocol

The BIPAMS study fidelity protocol addresses all five areas of the NIH BCC, including both fidelity protocol and monitoring plans that ensure uniformity among providers and replicability. Our fidelity protocol and monitoring plans are based on previous work by author BCW involving behavior change in spinal cord injury [18]. The intervention includes both BIPAMS and WellMS conditions with differences in contact frequency and format that require some group-specific strategies; however, where possible, strategies and monitoring plans are uniform between groups. All fidelity monitoring will be completed through the formal study period, as this will confirm implementation of protocols as intended. The five stages of fidelity fit linearly in the research process, with study design occurring prior to the intervention; provider training occurring prior to and during the intervention; and treatment delivery, receipt, and enactment occurring during the intervention phase. The frequency, NIH BCC areas, and data sources of the BIPAMS fidelity-monitoring plan are outlined in Table 2.

**Table 1:** Description of intervention components for the BIPAMS and WellMS conditions.

Intervention component		BIPAMS^a^	WellMS^b^
**Internet website**			
	Target	Physical activity	General wellness
	Primary source of intervention content	Previous research by principal investigator	National Multiple Sclerosis Society
	Theoretical underpinnings	Social cognitive theory	Social cognitive theory
	Interactive video courses, n	10	10
	Resource section	Yes	Yes
	Learn more section	Yes	Yes
	Physical activity tracker	Yes	No
	Forum	Yes	Yes
	Patient voices, n	24	10
	Weekly email announcements	Yes	Yes
	Weekly updates on the website	Yes	Yes
	Tips of the week	Yes	Yes
	News and events section	Yes	Yes
**One-on-one video calls**			
	Occurrence, n	13	9
	Semiscripted guide	Yes	Yes
	Adverse event reporting	Yes	Yes
**Other**			
	Pedometer	Yes	No
	Goal setting	Yes	Yes
	Log books/self-monitoring	Yes	Yes

^a^BIPAMS: Behavioral Intervention for Physical Activity in Multiple Sclerosis.

^b^WellMS: Wellness for Multiple Sclerosis.

**Table 2:** Overview of the study fidelity-monitoring plan.

Data source	Monitoring frequency	Areas of fidelity addressed				
		Study design	Provider training	Treatment delivery	Treatment receipt	Treatment enactment
Coaching call checklist	Monthly	Yes	Yes	Yes	No	No
Coaching call logs	Monthly	Yes	No	Yes	No	No
Auditing of coaching calls by expert	Weekly	Yes	Yes	Yes	Yes	No
Behavioral resource bank within treatment group	Quarterly	Yes	No	No	No	No
Review of participant website log-in	Weekly	No	No	No	Yes	Yes
Review of participant exercise log (BIPAMS^a^) or log book (WellMS^b^)	Weekly/monthly	No	No	No	Yes	Yes
Team meetings to discuss participant progress and protocol adherence	Weekly	Yes	Yes	Yes	Yes	Yes

^a^BIPAMS: Behavioral Intervention for Physical Activity in Multiple Sclerosis.

^b^WellMS: Wellness for Multiple Sclerosis.

### Fidelity of Study Design

The NIH BCC fidelity of study design area focuses on practices that ensure study procedures and implementation are in line with current theory and clinical processes. Study design fidelity goals include ensuring that conditions are congruent with relevant theory and practice, ensuring equivalent treatment dose within and across conditions, and planning for implementation setbacks.

The first study design goal includes the congruence of the conditions with relevant theory and practice. As such, both conditions include evidence-based SCT behavior-change strategies in the website components as well as one-on-one video calls. Importantly, the WellMS intervention focuses on wellness based on resources from the National Multiple Sclerosis Society, whereas BIPAMS focuses on physical activity based on previous research and clinical practice. The website content and coach training are consistent with SCT principles for behavior change, and these were efficacious in the phase II trial of BIPAMS [9]. Two fidelity strategies will be implemented to track the use of SCT during video calls. The first strategy involves checklists for each video call that focus on goal setting and self-efficacy to, for example, ensure the use of SCT principles. This strategy is carried out with every video call. In the second strategy, video calls will be randomly audited by an expert for a review of principles from SCT. Each coach will have one call per content week that will be audited by an expert in SCT and eHealth delivery (BCW and RWM). We define such an expert as a principal investigator on a current or previously funded eHealth behavioral intervention based on SCT. Auditing is done randomly each week in person (one call audited per coach); no coaching calls will be recorded in any phase of the study. Adherence to these fidelity measures will be actively monitored by the project coordinator through monthly audits of the checklists for each participant per coach (Multimedia Appendices 1 and 2). Further, there will be ongoing review of the website in order to ensure that all content is working and up to date. Table 3 provides further details on the resources and frequency of monitoring.

The second goal regarding the study design involves ensuring equal treatment dose within and between conditions. Both conditions include a standard video call schedule, although the frequency of these calls varies slightly between conditions (Table 3). Both conditions will receive a weekly reminder with updated website content and additional tips that align with the topic. Therefore, both conditions will have a standardized dose with slight variations between them. This is largely based on the differential content between conditions (ie, physical activity vs general health and wellness) and the desire for making the control condition credible, but not overwhelming in diverse content for participants. The monitoring plans include in-person auditing of random video calls by experts and within-team meetings. Full team meetings that include coaches from both groups will occur weekly, while intragroup meetings will occur during content weeks. Intragroup meetings include discussions of call notes and duration focused on similar dose (ie, coaching call length) within groups. We anticipate that call length and content will vary depending on participant needs, particularly initially, when participants are learning to use the technology and website, but generally, calls should last between 10 and 30 minutes.

The third study design goal involves a plan for implementation setbacks such as website problems or coaching-related illness or travel. Both BIPAMS and WellMS have multiple trained coaches to address this goal, which serves as backup during unanticipated or scheduled events. In the event of travel or illness, trained coaches will fill in for the missing coach and follow all procedures (check lists, expert audits, etc), and we will monitor the number of times coaches change or cover for one another.

### Fidelity of Provider/Coach Training

The NIH BCC fidelity-monitoring plan further includes strategies that address preparation for uniform delivery of treatment by providers/coaches. Behavioral health interventions often require training in new skillsets, content, and protocols. The coaches (ie, providers) for the BIPAMS and WellMS conditions will interact directly with the participants regarding content discussion, accountability, and goal setting. The strategies and monitoring plan for fidelity of provider/coach training are outlined in Table 4 with standard training between groups except for content-specific materials (ie, physical activity vs wellness strategies).

**Table 3:** Fidelity of study design strategies and monitoring plan for BIPAMS.

Goal	Description from NIH BCC^a^	Strategies used		Fidelity-monitoring plan	
		BIPAMS^b^	WellMS^c^	BIPAMS	WellMS
Ensure intervention is congruent with relevant theory and practice	Operationalize treatment to optimally reflect theoretical roots; precisely define variables most relevant to “active ingredients” of the intervention	Coaches use evidence-based behavior-change strategies during calls and messages (SCT^d^) Integration of behavior-change strategies into website (SCT)	Coaches use evidence-based behavior-change strategies during calls and messages (SCT) Integration of behavior-change strategies into website (SCT)	Monthly review of coaching call checklist Auditing of random selection of calls by an expert on content weeks Initial review of the website before beginning enrollment and quarterly audit of resources provided within website modules, resources, and learn more information sections	Monthly review of coaching call checklist Auditing of random selection of calls by an expert on content weeks Initial review of the website before beginning enrollment and quarterly audit of resources provided within website modules, resources, and learn more
Ensure equal treatment dose within and across conditions	Ensure equal treatment “dose” (measured by number, frequency, and length of contact) is adequately described and is the same for each subject within a particular treatment condition	Standard call schedule for all participants on weeks 1, 2, 3, 4, 5, 6, 7, 8, 11, 12, 13, 16, and 20 Weekly email reminder with updated website content	Standard call schedule for all participants at weeks 1, 2, 3, 6, 8, 12, 13, 16, and 20 Weekly email reminder with updated website content	Auditing of random selection of calls by an expert on content weeks Weekly meeting within the team to review materials and call duration logs	Auditing of a random selection of calls by an expert on content weeks Weekly meeting within the team to review materials and call duration logs
Plan for implementation setbacks	Address possible setbacks in implementations (eg, treatment providers dropping out)	Train multiple providers to ensure back up in the event of provider vacation, illness, or turnover	Train multiple providers to ensure back up in the event of provider vacation, illness, or turnover	Tracking log with the number of times providers change/cover and reason	Tracking log with the number of times providers change/cover and reason

^a^NIH BCC: National Institutes of Health Behavior Change Consortium.

^b^BIPAMS: Behavioral Intervention for Physical Activity in Multiple Sclerosis.

^c^WellMS: Wellness for Multiple Sclerosis.

^d^SCT: social cognitive theory.

**Table 4:** Fidelity of provider training strategies and monitoring plan for BIPAMS.

Goal and description from NIH BCC^a^		Strategies used		Fidelity-monitoring plan	
		BIPAMS^b^	WellMS^c^	BIPAMS	WellMS
**Standardized training**					
	Ensure that training is conducted similarly for all providers	Standardized protocols and training materials: Study design Behavior-change theory (SCT^d^) Fidelity protocol overview Data-collection procedures Data-quality procedures	Standardized protocols and training materials: Study design Behavior-change theory (SCT) Fidelity protocol overview Data-collection procedures Data-quality procedures	Provider training records	Provider training records
**Ensure provider skill acquisition**					
	Train providers to well-defined performance criteria	Role playing Mock delivery Coaching call checklists	Role playing Mock delivery Coaching call checklists	Provider training records Monthly audit of coaching call checklists	Provider training records Monthly audit of coaching call checklists
**Minimize “drift” in provider skills**					
	Ensure provider skills do not decay over time	Monitor random selection of coaching calls Weekly meetings with PI^e^ and intervention staff to discuss intervention strategies and resolve difficult situations as they arise Standardize training of all providers: Behavior-change theory training Mock delivery	Monitor random selection of coaching calls Weekly meetings with PI and intervention staff to discuss intervention strategies and resolve difficult situations as they arise Standardize training of all providers: Behavior-change theory training Mock delivery	Auditing of random selection of calls by an expert on content week Provider training records	Auditing of random selection of calls by an expert on content weeks Provider training records
**Accommodate providers differences**					
	Ensure adequate level of training in providers of difference skill level, experience, or professional background	Coaching call checklist Monitor random selection of coaching calls Standardized materials provided on website Standardized scripts for each call	Coaching call checklist Monitor random selection of coaching calls Standardized materials provided on website Standardized scripts for each call	Monthly audit of coaching call checklists Auditing of random selection of calls by an expert on content weeks Weekly meeting among providers to discuss material Weekly meeting among providers and with PI to discuss material	Monthly audit of coaching call checklists Auditing of random selection of calls by an expert on content weeks Weekly meeting among providers to discuss material Weekly meeting among providers and with PI to discuss material

^a^NIH BCC: National Institutes of Health Behavior Change Consortium.

^b^BIPAMS: Behavioral Intervention for Physical Activity in Multiple Sclerosis.

^c^WellMS: Wellness for Multiple Sclerosis.

^d^SCT: social cognitive theory.

^e^PI: principal investigator.

The first goal within the fidelity of provider/coach training is standardized training; this ensures that training is conducted similarly for all providers. All coaches must have a bachelor’s degree in Exercise Science, Psychology, Kinesiology, or a related health field. Both groups of coaches will be trained by the principal investigator on the study design, SCT, fidelity protocol, data collection, and data quality procedures. Provider training records (eg, meeting dates and time log) will be maintained by coaches across training goals in this area, and monthly auditing will be conducted by the project coordinator.

BIPAMS and WellMS coaches will be trained separately using well-defined performance criteria in order to ensure and enhance skill acquisition. Training is utilized as an essential tool that familiarizes coaches with intervention-specific content, behavior-change strategies, and comfort in communication via virtual media. First, coaches will be trained on condition-specific content by using the website and previous literature to ensure thorough knowledge of resources and content. Thereafter, new coaches will undergo SCT and behavior-change strategy training with the principal investigator and then transition into hands-on training. All coaches will complete role playing and mock delivery of all content, with feedback provided by the research team. Coaching call checklists will be used in mock deliveries and all calls, thereby ensuring that providers meet all the performance criteria. In the event of poor skill acquisition (ie, not meeting 100% of the auditing checklist criteria), the principal investigator will be notified, and coaches will undergo additional training.

Another goal of provider training is minimizing drift in provider skills. The one-on-one video calls in the BIPAMS study will be conducted for 6-month periods across four waves of participants; this underscores the importance of coaches to revisit materials weekly and ensure consistency in communication over time with each participant and across waves. Standardized training and auditing of random calls, as previously mentioned, will be utilized to control for drift in both groups. Coaches will utilize call scripts and checklists for every video call throughout the intervention, thereby providing approaches for self-monitoring, maintaining consistency, and recording any drift. Additionally, weekly meetings with the principal investigator and intervention staff to address difficult situations that arise will be critical. These meetings provide an opportunity to solve problems within and across conditions as a team in order to ensure the coaches are consistent within the content area and understand the issues that can arise between conditions.

Providers are often different in many ways including professional background, personality, and experience. To accommodate provider differences, the NIH BCC recommends that researchers ensure an adequate level of training among providers. To account for these differences, standardization of training, website content materials, and phone call scripts are included in both conditions to provide uniform treatments and interactions. Weekly meetings among team members (ie, coaches) and the principal investigator will be conducted to clarify and review materials throughout the training and implementation phases. Additionally, coaching call checklists and live monitoring of video calls by experts (BCW and RWM), as previously outlined, address this goal and keep providers focused on the intended active ingredients. Any specific questions addressed by providers in a unique manner will be discussed in weekly meetings among coaches, as participants often have similar experiences.

### Fidelity of Delivery of Treatment

Fidelity of treatment delivery focuses on ensuring the intervention is delivered as intended. Many of the concerns within delivery of treatment overlap with strategies for training and study design, including controlling for provider differences and adhering to created protocols; however, this area further addresses differences within treatment conditions and minimizes contamination (Table 5).

Both conditions have a unique team of coaches that focus on either BIPAMS or WellMS content; therefore, coaches only coach one condition. Auditing of random one-on-one video calls by experts (BCW and RWM) will be the primary strategy for controlling provider differences during the treatment-delivery phase of this study. These expert-audited video calls include a specific checklist of expected provider actions such as goal setting, website resource use, and content comprehension. The expert auditors will further provide feedback on the handling of unique questions and conversation topics that require reframing back to the week’s content and condition-specific goals. The expert auditors will not be blinded to groups due to necessary content checking such as asking about steps for BIPAMS and weekly content-specific goals for WellMS.

As mentioned previously, the BIPAMS and WellMS conditions differ in foci and content; however, both provider teams will meet together on a weekly basis to review content. Weekly in-person meetings to discuss website content and resources ensure that providers within each condition understand and deliver the intended materials. Additionally, coaching call logs will be reviewed to assess and address any differences in the dose or time spent on calls. All participants within each condition will receive the same materials on the website and have access to the same content. During the video calls, coaches will ask if participants reviewed all content and encourage participants to review materials missed as well as provide an overview of the topic.

**Table 5:** Fidelity of delivery of treatment strategies and monitoring plan for BIPAMS.

Goal	Description from NIH BCC^a^	Strategies used		Fidelity-monitoring plan	
		BIPAMS^b^	WellMS^c^	BIPAMS	WellMS
Control for provider differences	Monitor and control for subjects perceptions of nonspecific treatment effects (eg, warmth, credibility) across conditions	Monitor random selection of coaching calls	Monitor random selection of coaching calls	Auditing of random selection of calls by an expert on content weeks	Auditing of random selection of calls by an expert on content weeks
Reduce differences within treatment	Ensure that providers in the same condition are delivering the same intervention	Coaches meet weekly to discuss materials and discussion plans for calls	Coaches meet weekly to discuss materials and discussion plans for calls	Weekly meetings among providers to discuss material	Weekly meetings among providers to discuss material
Ensure adherence to treatment protocol	Ensure that treatments are being delivered in the way they were conceived with regard to content and dose	Coaching call checklists Coaching call logs and missed call protocol Equal resources provided within the condition	Coaching call checklists Coaching call logs and missed call protocol Equal resources provided within the condition	Monthly auditing of coaching call checklists Monthly auditing of coaching call logs and missed call protocol Quarterly review of website resources to ensure all materials are available for both treatment groups	Monthly auditing of coaching call checklists Monthly auditing of coaching call logs and missed call protocol Quarterly review of website resources to ensure all materials are available for both treatment groups
Minimize contamination between treatments	Minimize contamination	Train all staff on theory and physical activity interventions underlying the study Train staff on answering questions related to randomization and group allocation in an unbiased way Train staff to identify topics of cross-contamination (ie, diet, emotions, and secondary conditions)	Train all staff on theory, health, and wellness behaviors underlying the study Train staff on answering questions related to randomization and group allocation in an unbiased way Train staff to identify topics of cross-contamination (ie, physical activity and exercise)	Provider training records Auditing of random selection of calls by an expert on content weeks Tracking log-documenting instances of cross-contamination for each content week	Provider training records Auditing of random selection of calls by an expert on content weeks Tracking log-documenting instances of cross-contamination for each content week

^a^NIH BCC: National Institutes of Health Behavior Change Consortium.

^b^BIPAMS: Behavioral Intervention for Physical Activity in Multiple Sclerosis.

^c^WellMS: Wellness for Multiple Sclerosis.

Participants will be informed at the screening that the study is an RCT and that participants are randomly assigned into BIPAMS or WellMS conditions. A general overview of each condition will be provided to all participants per Institutional Review Board requirements to present necessary information to participants when they make an informed decision on participation; therefore, cross-contamination was critical to address. Once randomized, participants will be provided with the condition-specific website link and a unique username and password; the websites are hosted in separate locations (Multimedia Appendices 3 and 4). BIPAMS participants will create goals and receive content regarding increasing physical activity. WellMS participants will receive content on different topics each week including nutrition, gait, stress management, and sleep and will be encouraged to create goals that align with these content areas outside of physical activity. Providers will be trained to identify potential topics of cross-contamination; for example, a WellMS participant who sets a physical activity-related goal or a BIPAMS participant who sets a nutrition goal. Participants will be encouraged to set goals that align with the content of the BIPAMS or WellMS conditions, and coaches will be trained to answer questions in an unbiased way. A record and description of any cross-contamination instances will be kept by coaches.

**Table 6:** Fidelity of receipt of treatment strategies and monitoring plan for BIPAMS.

Goal	Description from NIH BCC^a^	Strategies used		Fidelity-monitoring plan	
		BIPAMS^b^	WellMS^c^	BIPAMS	WellMS
Ensure participant’s comprehension	Ensure that participants understand the information provided by the intervention	Web-based physical activity tracker that coaches can view Use of open-ended questions and participant-led goal setting/action planning	Print-based log book that providers will inquire about/receive during each chat Use of open-ended questions and participant-led goal setting/action planning	Weekly review of exercise tracker Participant reports during auditing of random selection of calls by an expert on content weeks	Inquiry about log book use at each chat Participant reports during auditing of random selection of calls by an expert on content weeks
Ensure participants ability to use cognitive skills	Make sure that participants are able to use the cognitive skills taught in the intervention (ie, reframing, problem solving, preparing for high-risk situations)	Use of open-ended questions and participant-led goal setting/action planning Narrative coaching notes	Use of open-ended questions and participant-led goal setting/action planning Narrative coaching notes	Auditing of random selection of calls by an expert on content weeks for coaches’ use of techniques and responses to participant questions Review of notes/questions during weekly intervention meetings	Auditing of random selection of calls by an expert on content weeks for coaches’ use of techniques and responses to participant questions Review of notes/questions during weekly intervention meetings
Ensure participants ability to perform behavioral skills	Make sure that participants able to use behavioral skills taught in the intervention (eg, relaxation, food diaries, cigarette-refusal skills)	Initial call to ensure receipt and use of training materials and ensure participants understand all aspects of the website	Initial call to ensure receipt and use of training materials and ensure participants understand all aspects of the website	Quarterly review of coach call logs for initial call Review of participant log-in throughout the study	Quarterly review of coach call logs for initial call Review of participant log-in throughout the study

^a^NIH BCC: National Institutes of Health Behavior Change Consortium.

^b^BIPAMS: Behavioral Intervention for Physical Activity in Multiple Sclerosis.

^c^WellMS: Wellness for Multiple Sclerosis.

### Fidelity of Receipt of Treatment

Fidelity of treatment receipt involves strategies and monitoring of a participant’s ability to understand and adopt treatment-related behavioral skills and cognitive strategies. Working with people with MS can make identifying receipt of treatment issues challenging because of cognitive impairment. This is important, as cognitive impairment is not an exclusion criterion. The research team created strategies and a monitoring plan to document receipt of treatment based on the NIH BCC best practices and goals by assessing and optimizing participant comprehension of materials (Table 6). Receipt of treatment further encompasses the degree to which participants demonstrate knowledge of and ability to use treatment skills.

Participants in both conditions will complete an initial, one-one-one call with a behavioral coach and confirm receipt of intervention materials and instructions on access and use of the online platforms for content and video calls. The initial call plays a role in ensuring participants are able to access materials and use the information taught in the intervention. The use of video calls throughout the intervention will provide the coaches with opportunities to assess comprehension of materials using visual cues (eg, facial expressions and body language) and real-time interactions to inquire about participants accessing website and reviewing modules, resources, and patient videos. The project coordinator will also review participant website log-in activity (Multimedia Appendix 5) weekly throughout the study to document when participants access the website and the study materials on an ongoing basis.

This intervention further depends on participants’ comprehension and ability to utilize digital media in delivering content and tracking goals. Participants in the BIPAMS condition will be provided a pedometer and log steps in a journal daily (Multimedia Appendix 6) and transfer step counts into the website at the end of each week. This will allow the research team to monitor receipt of treatment on a weekly basis through the website. Participants in the WellMS condition will be asked to log goals and notes in a paper-based log book (Multimedia Appendix 7) on a daily basis, but not to transfer them into the website. The research team will document whether the log books are used weekly and ask participants to send the log book after the 6-month intervention period.

**Table 7:** Fidelity of enactment of treatment strategies and monitoring plan for BIPAMS.

Goal	Description from NIH BCC^a^	Strategies used in BIPAMS^b^	Strategies used in WellMS^c^	Fidelity-monitoring plan for BIPAMS	Fidelity-monitoring plan for WellMS
Ensure participants use cognitive skills	Ensure that participants actually use the cognitive skills provided in the intervention in appropriate life settings	Review of coaching calls	Review of coaching calls	Monthly tracking of coaching notes	Monthly tracking of coaching notes
Ensure participants use behavioral skills	Ensure that participants actually use the behavioral skills provided in the intervention in appropriate life settings	Review of use in calls Web-based exercise tracker that coaches can view Review of participant weekly log-ins	Review of use in calls Print-based log book that providers will inquire about/receive at each chat Review of participant weekly log-ins	Monthly tracking of coaching notes Weekly website review by coaches Study coordinator review of website participant log-in throughout the study	Quarterly review of coach call logs for the initial call Monthly tracking of coaching notes Study coordinator review of website participant log-in throughout the study

^a^NIH BCC: National Institutes of Health Behavior Change Consortium.

^b^BIPAMS: Behavioral Intervention for Physical Activity in Multiple Sclerosis.

^c^WellMS: Wellness for Multiple Sclerosis.

Based on SCT, participants will be encouraged to be the leaders of their behavior change, with coaches available to provide knowledge and support. Provider training includes established practices in participant-driven goal setting and action planning that use open-ended questions, providing participants the opportunity to voice concerns and autonomy in leading the conversation. This strategy will allow the research team to ensure participants comprehend materials and are capable of using cognitive skills for creating unique goals. Narrative coaching notes for each video call and auditing of calls by an expert will be set in place to monitor the use of these strategies and participant reports.

### Enactment of Treatment

The fifth area highlighted by the NIH BCC is enactment of treatment described as strategies aimed at monitoring and improving participant ability to perform treatment-related behavioral skills and cognitive strategies in relevant real-world settings. This area is particularly important in a longitudinal behavior change study like BIPAMS, as outcomes are focused on the degree to which participants apply skills learned as part of daily life. The goals for enactment of treatment focus on ensuring participants use both the cognitive and behavioral skills learned (Table 7).

Use of cognitive skills provided in the intervention in appropriate life settings will be documented and assessed in narrative coaching notes. Check-in video calls with participants will occur each week that new content is presented on the condition-specific website. The video calls will provide coaches with rich qualitative data regarding the use of cognitive skills learned as part of the conditions. Coaches will assess the use of behavioral skills during video calls through specific questions outlined in the coaching call scripts. As previously mentioned, BIPAMS and WellMS conditions have unique logging resources for self-monitoring new behaviors or tracking goals that will provide an additional means of monitoring treatment enactment. Participant website log-ins will be tracked in order to monitor enactment of new behaviors associated with follow through on intervention responsibilities such as review of content and weekly tips/updates.

### Data Analysis

The NIH BCC treatment fidelity workgroup does not provide guidance on the analysis of treatment fidelity data [13]. Previous researchers have incorporated high versus low fidelity in study design, treatment, and data analysis [14,15]; however, they do not provide clear analysis plans for fidelity metric data. Therefore, we have created a data-analysis plan to describe each fidelity metric within and across BIPAMS and WellMS conditions using descriptive statistics, specifically mean (SD), percentages, and frequency counts (range).

## Results

Enrollment began in February 2018 and will conclude in September 2019. Intervention delivery will conclude in March 2020. Data analysis and full study results are expected in the summer of 2020.

## Discussion

BIPAMS is a phase III RCT of an internet-delivered behavioral intervention based on SCT and principles of e-learning for increasing physical activity among persons with MS. The BIPAMS study includes an intervention condition (BIPAMS) and control condition (WellMS) delivered through internet websites and supported by video calls with coaches trained in SCT and associated behavior-change strategies. The primary outcome is objectively measured MVPA. The secondary outcomes are self-report physical activity, walking mobility, cognitive function, fatigue, depression, anxiety, sleep quality, pain, and change in disability. The tertiary outcomes are self-efficacy, outcome expectations, goal setting/planning, and facilitators/impediments. Another set of outcomes is treatment fidelity for optimizing and assessing the rigor and reproducibility of the BIPAMS intervention in MS. This paper highlights our approach for rigor and reproducibility when reporting the effects of the BIPAMS intervention condition on primary, secondary, and tertiary study outcomes.

We applied the NIH BCC goals and applicable strategies for establishing the fidelity of this phase III trial. The NIH BCC fidelity-monitoring scheme includes five areas, and we included all five areas for complete mapping and monitoring of the fidelity of the BIPAMS study. Strategies for ensuring and monitoring fidelity in the intervention will include standardized scripts, standardized call schedules, comprehensive provider training in content and theory, ongoing phone call monitoring by a trained expert, monitoring website usage, and monitoring of self-monitoring strategies. This protocol is specific for an eHealth/e-learning physical activity intervention for persons with MS; however, the underlying strategies and themes are applicable for other studies. This report provides guidance for other researchers conducting phase III RCTs that are focused on evaluating the validity of behavioral interventions; this is particularly important as interventions in phase III are intended to test effectiveness and be widely disseminated for clinical or practical applications.

Some interventions may benefit from using the modified NIH BCC guidelines that align with associated study objectives. One group of researchers previously used the NIH BCC model to create an approach to address fidelity in the context of rehabilitation research [19]. This involved collapsing the five areas from the NIH recommendations into three focal areas, namely, intervention and study design, resourcing, and implementation. These broader definitions are applicable to clinical rehabilitation research, wherein clinical staff may be incorporating research into existing practice, thereby removing control over provider training and therapy/intervention protocols. For example, one group of researchers conducted a physical therapist-led intervention and reported that the NIH BCC goals did not precisely match the unique circumstances that arose within a clinical setting [20]. That protocol paper included several iterations to create an implementation-specific protocol utilizing the NIH areas and further provided a model for researchers interested in validating unique, intervention-specific fidelity practices. Although fidelity protocols are pivotal in replicating studies, an additional resource that can assist future evidence-based intervention research is the Template for Intervention Description and Replication (TIDieR) [21]. Based on previous research, we assert that behavioral intervention studies should include fidelity protocols and monitoring as well as use of the TIDieR checklist in order to move the field forward to address threats to validity and provide clarity for replicating studies.

Fidelity protocols and monitoring provide a quantifiable means of monitoring the rigor of the BIPAMS study. Previous iterations of the study demonstrated preliminary efficacy for BIPAMS in improving MVPA and secondary outcomes (ie, fatigue severity, physical impairment, depression, and anxiety) [9]. This iteration of BIPAMS includes an intervention and control condition that receives an intervention; therefore, standardization between conditions and coaches is essential. Our focus on disseminating a rigorous fidelity-monitoring protocol using the NIH BCC recommendations represents a pivotal next step in reporting strategies that test the true replicability and effectiveness of the BIPAMS intervention. Such a protocol will provide a foundation for fidelity practices in physical activity RCTs for people with MS.

The BIPAMS study fidelity protocol has some limitations. The BIPAMS and WellMS conditions (Table 1) include different contents and overall dose, as BIPAMS includes 13 video calls and WellMS includes 9 video calls. Specifically, this contributes to barriers when comparing and interpreting fidelity between two active conditions like BIPAMS and WellMS that share primary components but do not mirror each other perfectly. Both conditions include self-monitoring components, but BIPAMS is performed on paper and uploaded virtually, whereas WellMS only includes a paper log. However, the theoretical underpinnings of the conditions are consistent with previous iterations and the dose is equal within groups (ie, all BIPAMS participants receive the same content and number of calls). The interventions were previously designed and the evidence-based materials were kept in a validated format, wherever possible. Our study does not directly assess cognitive skills or disease severity and participants may have cognitive deficits that contribute to receipt and enactment of treatment; however, personalization based on the severity of disease is addressed in one-on-one video calls with behavioral coaches. Lastly, there is a threat to assessing fidelity in all studies wherein participants may provide socially desirable responses to behavioral coaches that do not accurately reflect engagement in intervention. Despite those limitations, fidelity protocols and monitoring provide the opportunity for a research team to evaluate where differences due to intervention content versus issues with internal validity may influence outcomes and interpretations.

The NIH BCC treatment fidelity workgroup provides recommendations for strategies to incorporate fidelity practices in behavior-change interventions. The BIPAMS phase III RCT integrated strategies in study design, provider training, delivery of treatment, receipt of treatment, and enactment of treatment to preserve internal validity and enhance external validity. The goal of the BIPAMS study is to create and test an evidence-based behavior-change intervention that can be used in the community and widely benefit people with MS. The treatment-fidelity procedures outlined in this report provide a model for other researchers to create studies in all phases and fit within the aims and priorities of translational research.

## Appendix

Multimedia Appendix 1 Behavioral Intervention for Physical Activity in Multiple Sclerosis audit checklist.

Multimedia Appendix 2 Wellness for Multiple Sclerosis audit checklist.

Multimedia Appendix 3 Behavioral Intervention for Physical Activity in Multiple Sclerosis website homepage.

Multimedia Appendix 4 Wellness for Multiple Sclerosis website homepage.

Multimedia Appendix 5 Weekly website log-ins.

Multimedia Appendix 6 Behavioral Intervention for Physical Activity in Multiple Sclerosis logbook page example.

Multimedia Appendix 7 Wellness for Multiple Sclerosis logbook page example.

## Abbreviations

BIPAMS: Behavioral Intervention for Physical Activity in Multiple Sclerosis
eHealth: electronic health
E-Learning: electronic learning
MS: multiple sclerosis
MVPA: moderate-to-vigorous physical activity
NIH BCC: National Institutes of Health Behavior Change Consortium
PAR-Q: Physical Activity Readiness Questionnaire
PI: principal investigator
RCT: randomized controlled trial
SCT: social cognitive theory
TIDieR: Template for Intervention Description and Replication
WellMS: Wellness for Multiple Sclerosis
