# Supplementary figures and images for: eHealth-Based Behavioral Intervention for Increasing Physical Activity in Persons With Multiple Sclerosis: Fidelity Protocol for a Randomized Controlled Trial
Source: JMIR Res Protoc. 2019 Mar 1;8(3):e12319. doi: 10.2196/12319 (PMC6418483; doi:10.2196/12319)

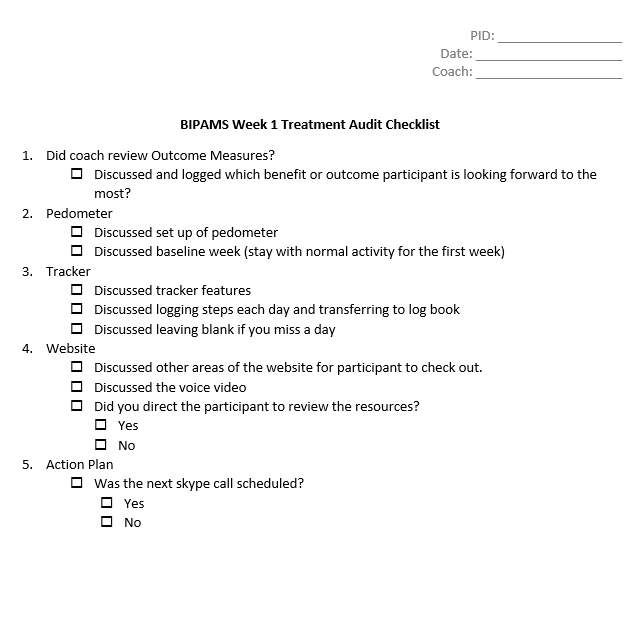

Supplement: Multimedia Appendix 1 [file resprot_v8i3e12319_app1.PNG]

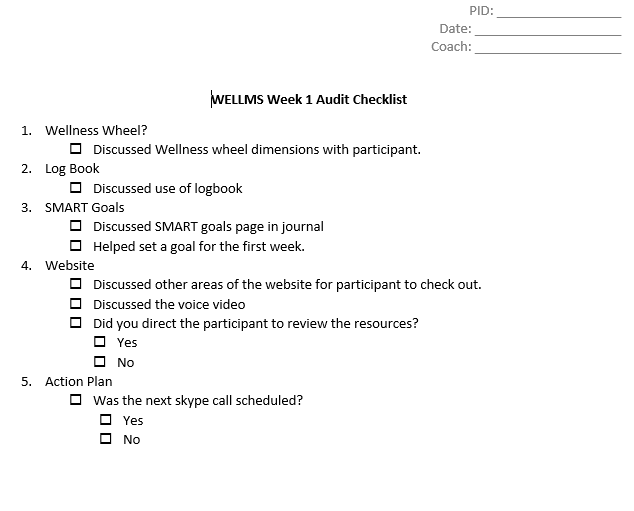

Supplement: Multimedia Appendix 2 [file resprot_v8i3e12319_app2.PNG]

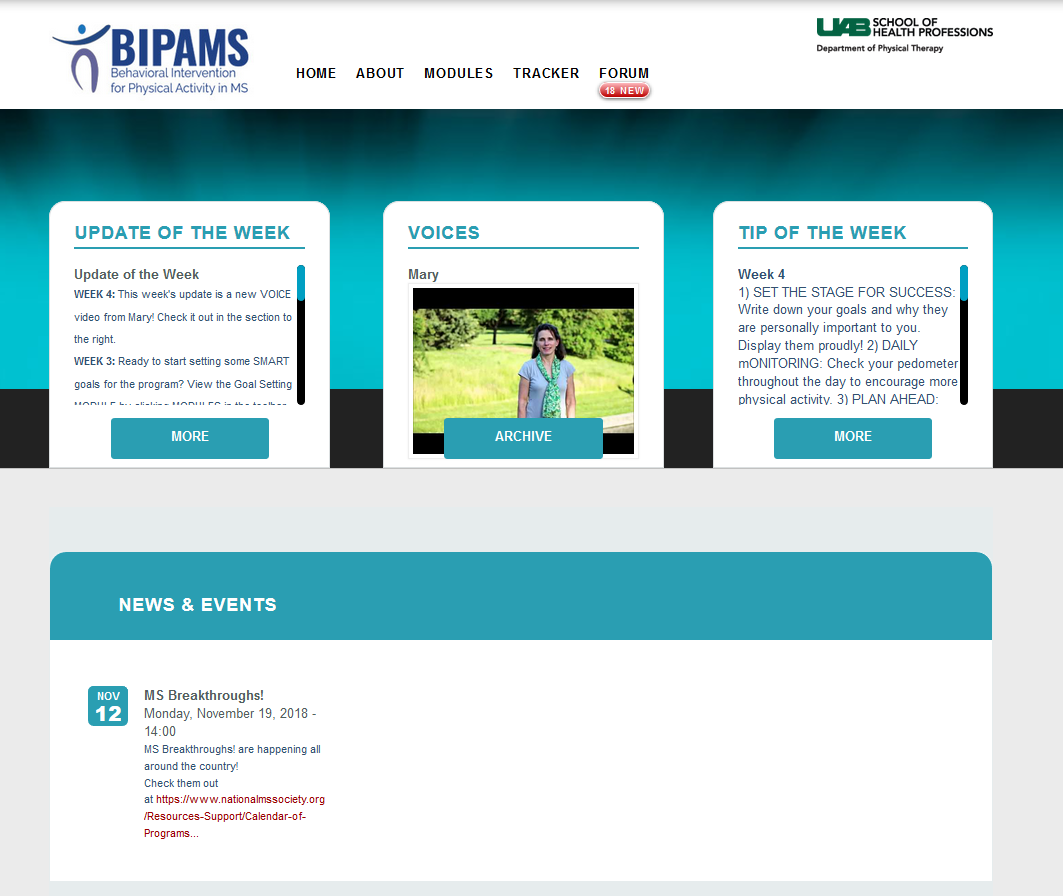

Supplement: Multimedia Appendix 3 [file resprot_v8i3e12319_app3.PNG]

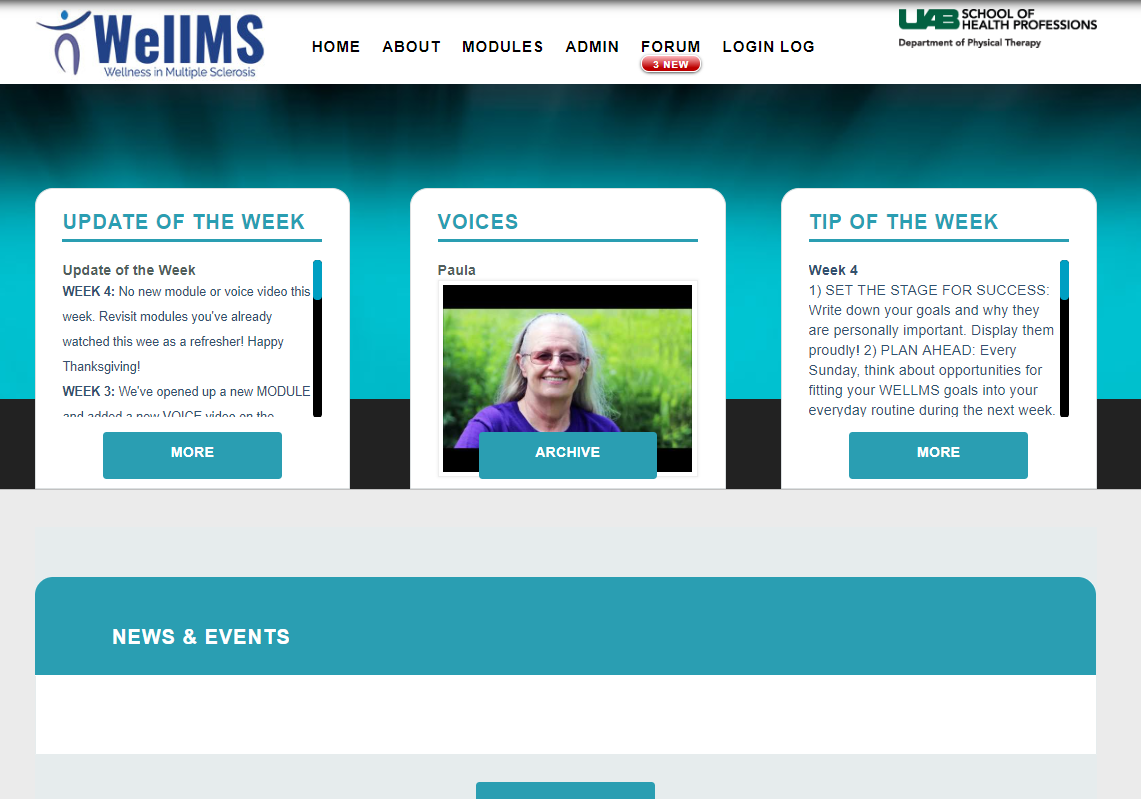

Supplement: Multimedia Appendix 4 [file resprot_v8i3e12319_app4.PNG]

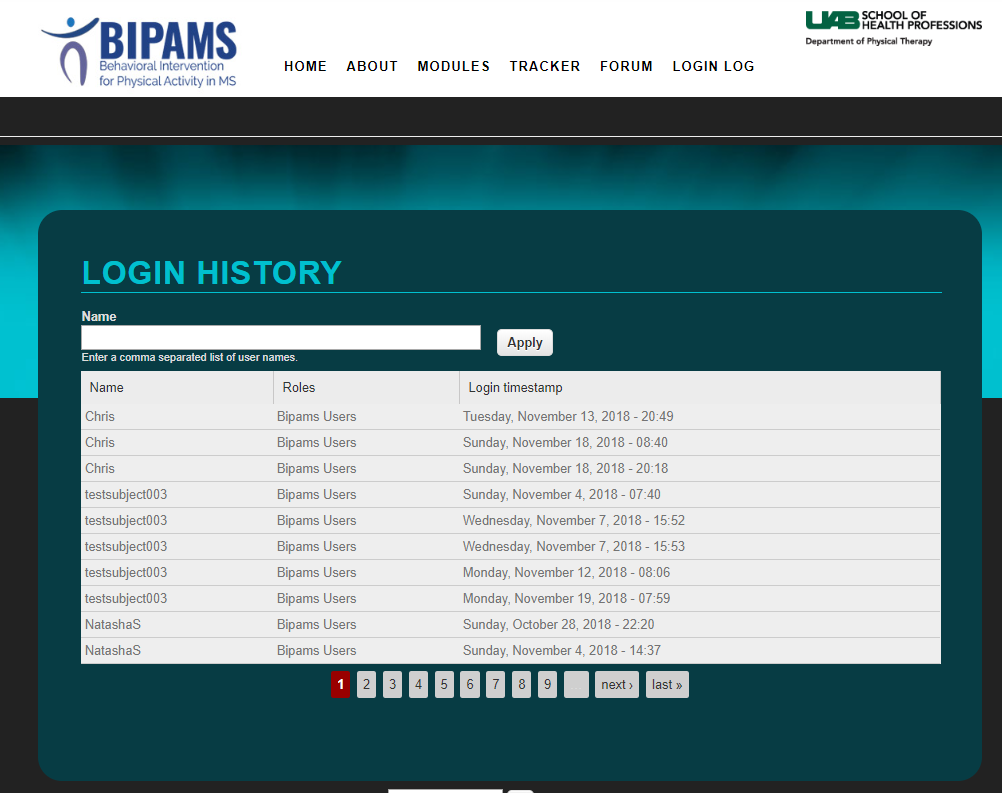

Supplement: Multimedia Appendix 5 [file resprot_v8i3e12319_app5.PNG]

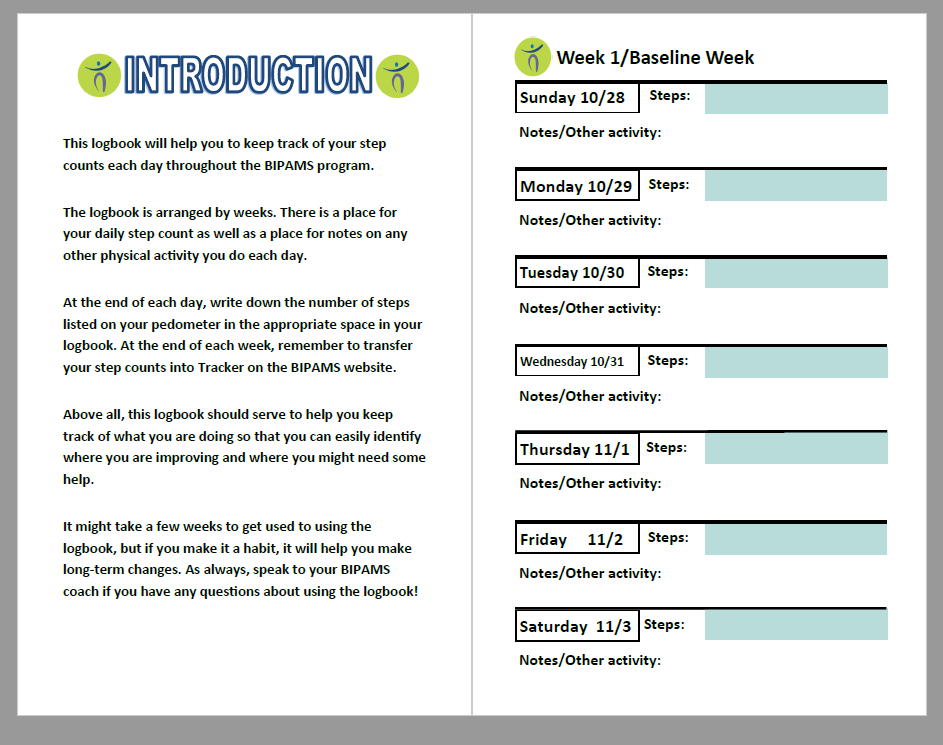

Supplement: Multimedia Appendix 6 [file resprot_v8i3e12319_app6.PNG]

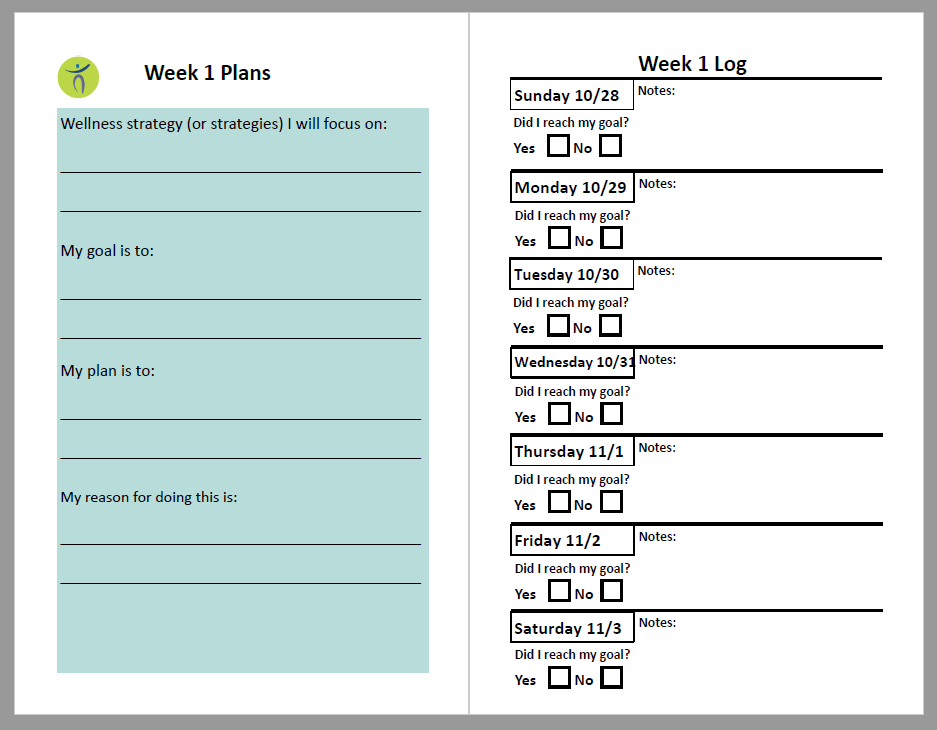

Supplement: Multimedia Appendix 7 [file resprot_v8i3e12319_app7.PNG]
